# Mental health-related help-seeking and the role of HIV care providers: A qualitative study with people with HIV in Cameroon

**DOI:** 10.1371/journal.pgph.0004597

**Published:** 2025-05-15

**Authors:** Angela M. Parcesepe, Kathryn E. L. Grimes, Peter Vanes Ebasone, Anastase Dzudie, Milton L. Wainberg, Eric Pefura, Marcel Yotebieng, Kathryn Anastos, Denis Nsame, Annereke Nyenti, Denis Nash

**Affiliations:** 1 Gillings School of Global Public Health, Department of Maternal and Child Health, University of North Carolina at Chapel Hill, Chapel Hill, North Carolina, United States of America; 2 Carolina Population Center, University of North Carolina at Chapel Hill, Chapel Hill, North Carolina, United States of America; 3 Gillings School of Global Public Health, Department of Health Policy and Management, University of North Carolina at Chapel Hill, Chapel Hill, North Carolina, United States of America; 4 Clinical Research Education Networking and Consultancy, Yaounde, Cameroon; 5 Department of Psychiatry, Columbia University, and New York State Psychiatric Institute, New York, New York, United States of America; 6 Jamot Hospital, Yaounde, Cameroon; 7 Department of Medicine, Albert Einstein College of Medicine, Bronx, New York, United States of America; 8 Departments of Medicine and Epidemiology & Population Health, Albert Einstein College of Medicine, Bronx, New York, United States of America; 9 Bamenda Regional Hospital, Bamenda, Cameroon; 10 Limbe Regional Hospital, Limbe, Cameroon; 11 Institute for Implementation Science in Population Health, City University of New York, New York New York, United States of America; Chinese Academy of Medical Sciences and Peking Union Medical College, CHINA

## Abstract

Mental health disorders are common among people with HIV (PWH), and the overwhelming majority of PWH with mental health disorders do not receive evidence-based mental health care. One promising strategy to increase access to evidence-based mental health care for PWH is to integrate mental health screening and treatment for common mental disorders and unhealthy drinking into HIV care. However, little is known about how PWH view the role of HIV care providers in supporting their mental health or their experiences with mental health-related help-seeking. We conducted in-depth interviews with 30 PWH at three HIV treatment centers in Cameroon. Interviews were conducted in French or English. French transcripts were translated to English for analysis. Thematic analysis was used to identify key factors that influenced mental health-related help-seeking and respondents’ preferred roles of HIV care providers. Overall, participants reported that they were less likely to seek support from medical providers for mental health compared to physical health needs. Previous positive contact with providers facilitated mental health help-seeking intentions among participants. Health facility characteristics, including limited provider availability, concerns about privacy and confidentiality, and high clinic volume were noted as barriers to mental health help-seeking at HIV facilities. Participants consistently reported that they desired positive, caring interactions with HIV care providers and clinic staff and noted that providers could play a critical role in helping PWH accept their HIV diagnosis and address HIV-related stigma and material needs. Overall, this sample of PWH in Cameroon reported that mental health-related help-seeking was limited in HIV care settings, but identified strategies that have the potential to enhance mental health related-help seeking in HIV care settings and may improve the mental health of PWH. The extent to which such strategies enhance mental health help-seeking and improve mental health should be evaluated.

## Introduction

Mental health disorders are common among people with HIV (PWH) globally, including in Cameroon [1–3]. It has been estimated that approximately 50% of PWH globally have one or more mental health disorders [4]. Among PWH in Cameroon, 20%, 15%, and 12% of PWH initiating HIV care reported moderate to severe symptoms of depression, PTSD, and anxiety, respectively [1]. Untreated mental health disorders have been associated with suboptimal outcomes throughout the HIV care continuum, including entry into HIV care with advanced HIV, suboptimal adherence to antiretroviral therapy (ART), and viral non-suppression [1,5,6]. Receipt of adequate, evidence-based mental health care has been associated with improved mental health and quality of life and may improve HIV care outcomes [7–10]. However, most people with mental health disorders across global settings do not have access to evidence-based mental health care. Approximately 90% of people with mental health disorders in low- or middle-income countries do not receive adequate mental health care [11,12]. Despite increased awareness of the needs and benefits of evidence-based mental health care, the global mental health treatment gap persists, including at HIV treatment centers throughout sub-Saharan Africa [13].

One promising strategy to increase access to evidence-based mental health care for PWH is to integrate mental health screening and treatment into HIV care settings [14–16]. Depending on the psychiatric diagnoses and severity of symptoms, integration of or linkage to mental health services into HIV care settings is recommended by the World Health Organization and guidelines for such integration or linkage to care have been developed [17,18]. Research suggests that evidence-based mental health interventions can be effectively integrated to treat common mental disorders (e.g., depression, anxiety, PTSD) into HIV care and delivered by lay health workers [7]. For example, a randomized controlled trial found that interpersonal therapy (IPT) delivered by nonspecialists at an HIV clinic in Kenya was associated with reductions in depression and PTSD [19]. Similarly, group IPT integrated into HIV care in Senegal was found to acceptable, feasible, and associated with improvements in depressive symptoms [20]. Task-shared cognitive behavioral therapy (CBT) has been associated with improvement in depression and ART adherence among PWH in South Africa [21]. Despite such evidence, integration of mental health screening and treatment for common mental disorders into HIV clinic settings remains limited [22,23]. Further, little is known of the perspective of PWH about their experience with mental health-related help-seeking from HIV care providers and their preferences for the role of HIV care providers in supporting their mental health. Better understanding of mental health help-seeking among PWH can inform the development, implementation, and acceptability of strategies to integrate mental health screening and treatment for common mental disorders into HIV care settings in Cameroon and beyond. The objective of this paper is to better understand the experience of mental health help-seeking among PWH in HIV care settings and ways in which HIV care providers can best support the mental health needs of PWH in Cameroon.

## Methods

The data used in this analysis come from a study that sought to better understand mental health and substance use disorders among PWH in low-resource settings. Quantitative and qualitative data were collected at three public urban HIV treatment facilities in Cameroon. The study sites were selected due to their participation in the Central Africa International epidemiology Database to Evaluate AIDS (CA-IeDEA) consortium [24]. Cameroonian study staff collected data in-person with PWH aged 21 or older newly enrolling in HIV care at one of the three participating facilities. All data were collected in French or English in a private setting at the facility.

### Ethics statement

All participants provided written informed consent. The Institutional Review Board at the University of North Carolina at Chapel Hill (Protocol 21–1175) and the National Ethical Committee of Research for Human Health in Yaoundé, Cameroon provided ethical approval for this study.

### Data collection

In-depth interviews were conducted with 30 PWH who had previously participated in a quantitative study about mental health among PWH in Cameroon [1]. In-depth interviews focused on why PWH might experience mental health problems, what makes it easier or harder to see a health care provider when experiencing mental health problems, and what health care providers need to know about caring for PWH who experience mental health problems (S1 Text). Convenience sampling was used to identify PWH willing and able to return to the clinic within approximately four weeks of completion of the follow-up quantitative survey [25]. Research assistants identified potential participants based on their meaningful engagement in prior research activities and their willingness and comfort discussing sensitive topics during prior data collection activities. Thirty total interviews (10 per facility) were deemed appropriate due to the relative homogeneity of the sample and narrow study objectives and is consistent with research that has found that 9–17 interviews are typically sufficient to reach saturation [26]. Interviews were conducted between October 1 and December 15, 2021, and were audio recorded. Due to the poor quality of one interview recording, 29 interviews were used for this analysis.

All of the PWH who participated in the qualitative interviews had previously participated in a baseline survey at the time of HIV care initiation (between June 2019 – March 2020) and a follow-up survey (between October 2021 and December 2021). These surveys included questions on sociodemographic characteristics, depression, anxiety, PTSD, and alcohol use. In these surveys, depressive symptoms were assessed with the Patient Health Questionnaire-9 (PHQ-9). Scores of 10 or greater were categorized as moderate or severe depressive symptoms [27]. Anxiety symptoms were measured with the Generalized Anxiety Disorder (GAD)-7 item screener. Scores of 10 or greater were categorized as moderate or severe anxiety symptoms [28]. PTSD symptoms were measured with the PTSD Checklist for DSM-5 (PCL-5) [29]. Scores of 31 or greater were categorized as probable PTSD [30]. Alcohol use was measured using the Alcohol Use Disorders Identification Test (AUDIT) [31]. Scores of 7 or greater for women and 8 or greater for men were categorized as unhealthy drinking. A dichotomous variable was created to categorize those with and without symptoms of depression, anxiety, PTSD, or unhealthy drinking at baseline or follow up interviews. This information was included when sharing participant quotes.

### Data analysis

Audio recordings were transcribed in the language in which the interview was conducted (French or English). A native French speaker transcribed French interviews and then translated them into English. All transcripts were compared to the original audio recordings, and translations were checked for accuracy. The finalized English transcripts were used for this analysis.

Interview data were assessed using thematic analysis. Transcripts were read multiple times to become familiar with the data. Using a subset of six randomly selected transcripts (two per site), KELG generated a list of more than 80 initial inductive codes and developed a draft codebook in Microsoft Excel. The codebook was revised through team discussions to streamline similar codes and clarify definitions. KELG and AMP blindly double coded three randomly selected transcripts (one per site), discussed discrepancies, arrived at consensus for code application, and modified the codebook as appropriate. KELG coded the remaining transcripts, meeting regularly with AMP to discuss changes to the codebook and how codes were applied. When a new code was developed, previously coded transcripts were reviewed to ensure consistent application across all data. The final codebook included 61 unique codes. All coding was completed using Dedoose software [32].

Following coding completion, excerpts were exported from Dedoose into Excel workbooks and organized by parent code. These Excel workbooks included demographic information (sex, age, reported mental health [MH] symptoms), clinic, code applications, and memos written throughout the coding process. KELG reviewed excerpts grouped by child code and wrote narrative summaries to search for themes in the data, exploring potential differences based on demographic information or clinic. Using the child code summaries, KELG wrote an overall summary for each parent code. KELG and AMP met weekly during writing to review and refine emerging themes.

## Results

### Participant characteristics

A total of 29 qualitative interviews were used for this analysis. A majority (69%) of interviewed participants were women with a mean age of 38 (range 22–73). Approximately half (52%) reported unhealthy drinking or moderate or severe symptoms of depression, anxiety, or PTSD in the baseline or follow-up surveys.

### Mental health help-seeking experiences in HIV care settings

#### Formal help-seeking for physical rather than mental health needs.

Participants reported that they primarily viewed their HIV care providers as available to address their physical and HIV-related health needs (Table 1). Several participants noted that they would be reluctant to seek out their HIV care provider for mental health needs or share mental health concerns with these providers. Several participants shared they simply did not visit the doctor when they felt sad. Participants explained that they preferred to handle such issues alone or with a friend and did not believe a doctor would be able to help them with mental health concerns. Some participants reported that they *“need[ed] to be a little [physically] sick to go see to the doctor” (Male, 37, reported mental health [MH] symptoms)*, only reaching out to formal sources of support if they were experiencing an issue related to their physical health. Others mentioned that *“the shame to sit in front of someone and ask for help, financial, emotional, whatever” (Female, 36, MH symptoms not reported)* was a barrier to seeking help.

**Table 1:** Key Themes related to mental health-related help-seeking among PWH in Cameroon.

Research Aim	Theme	Description
To better understand the experience of mental health help-seeking among PWH in HIV care settings	Formal help-seeking for physical rather than mental health needs	Participants reported that they primarily viewed their HIV care providers as available to address their physical and HIV-related health needs rather than mental health needs.
		Participants emphasized their commitment to seeking help for physical health needs regardless of their mental health status.
	Interpersonal interactions with HIV care providers	HIV care provider demeanor influenced participants’ mental health help-seeking from HIV care providers.
	Health facility characteristics	Knowing providers would be available increased participants’ confidence they would receive needed care if they visited the facility for mental health support.
		Privacy, high clinic volumes, long wait times, and challenges with patient flow were barriers to mental health help-seeking.
	Economic vulnerability, transportation-related barriers, and mental health help-seeking	Limited transportation and financial resources were barriers to healthcare access.
		Economic vulnerability (e.g., unemployment, limited financial resources) impacted participants’ mental health, and participants’ mental health affected their financial stability.
Strategies through which HIV care providers can support the mental health needs of PWH in Cameroon	Treat clients with “love and care”	Participants wanted to “feel at home” when receiving HIV care and many described the importance of providers listening to patients and asking questions to learn how they can best support their patients.
		HIV care providers were trusted sources of social support, with some characterizing their providers as a “second family” or someone who understood them more than anyone else.
	Promote acceptance of one’s HIV status	HIV care providers played an important role in helping PWH accept their HIV status. Acceptance of one’s HIV status influenced mental health.
	Economic empowerment and material support	Participants frequently discussed a desire for HIV clinics to provide opportunities for economic empowerment and to address their material needs.
		Participants indicated receiving direct financial support could help them meet a variety of needs, such as purchasing food for themselves or their families, paying their children’s education expenses, or paying for transportation to health care facilities.

Half of participants (15/29) reported that they viewed their HIV care provider’s role as limited to managing their physical health and did not view their HIV care provider as a potential resource for their mental health needs or concerns. Some participants (n = 3) viewed HIV care providers’ role as strictly limited to helping manage their physical health, indicating that providers just *“need to give me my medication, right? That’s it” (Female, 36, MH symptoms not reported)* and that *“they check that my blood pressure is normal. What else should I explain about myself?” (Female, 73, reported MH symptoms).* One participant reported that their provider did not ask them questions about their mental health in the same way they did about their physical health which informed their view that their provider was not a resource for their mental health needs. Most participants discussed their HIV care provider’s role as narrowly focused on managing HIV care and treatment – specifically *“mak[ing] sure that I take my drugs very well” (Female, 42, reported MH symptoms).*

Many participants drew parallels between their physical and mental health and emphasized their commitment to seeking help for physical health needs regardless of their mental health status. One participant described, *“If I’m sick, I don’t care, no matter how I’m sad, I run and see the doctor” (Female, 42, reported MH symptoms).* This commitment extended to attending routine HIV appointments. Ensuring they took ART as prescribed and attended routine HIV care appointments were the most frequently mentioned ways participants reported taking care of their physical health. Participants described the relationship between physical and mental health, explaining they felt “disturbed,” uncomfortable, or angry when they skipped or missed ART doses or were unable to manage opportunistic infections. Attending to physical health through ART adherence reduced stress and helped participants “feel comfortable” with their status:

*“So, when you follow the rules, when you take your medication on time, you get by well, without any problems, I think. For example, I, as you are seeing me in front of you, am bearing it and it doesn’t stress me out. I don’t have any stress, I don’t have any problems.” (Male, 62, MH symptoms not reported)*.

#### Interpersonal interactions with providers.

Despite viewing HIV care providers as primarily a resource for their physical health needs, participants across all facilities emphasized that positive, ongoing interpersonal interactions with HIV care providers facilitated mental health help-seeking from these providers. Being treated well by providers and staff when receiving HIV care instilled trust and confidence that seeking help for mental health needs would be well-received by providers and staff. One participant explained: *“When I feel sad […] it makes it very easy for me to see the doctor because they treat me with love and care” (Female, 46, reported MH symptoms).* Similarly, some participants reported that it was difficult to seek help for mental health concerns if they did not have a close relationship with their provider. Some participants recounted negative experiences, reporting that providers could be “very rude” when interacting with them. One participant described how being treated poorly by providers influenced their willingness to seek help:

*“When you asked me this question: when you have a problem, do you see the doctor? Well, it depends on the behavior, on how you treat people. If you are nice, if you know how to welcome and how to give people advice, if you smile at people, it invites them to come and see you. But if you are not nice, who will come to see you? Nobody.” (Female, 36, MH symptoms not reported)*

In addition to supporting formal help-seeking for mental health concerns, provider demeanor was associated with the extent to which participants felt they could be open with their providers when communicating their needs and concerns. If participants perceived the providers as kind, they felt they could open up and “talk freely,” which was deemed essential for improving general health and well-being and fostering mental health help-seeking.

#### Health facility characteristics that influence mental health help-seeking.

Participants emphasized the importance of provider availability in encouraging mental health help-seeking at HIV care settings. Knowing providers would be available increased participants’ confidence they would receive needed care if they visited the facility for mental health support:

*“Knowing that the doctor is always available to attend to you or to attend to me is one facility that maybe I will have assurance—or confidence—that okay, whenever I’m feeling sad I know if I go to the hospital or if I’m going to see my counselor, I know him or her is going to be there ready to listen to me or attend to me.” (Female, 22, reported MH symptoms)*.

Participants recounted experiences coming to the HIV clinic to find that their provider was unavailable. Contrastingly, other participants appreciated that their HIV care provider would make themselves available outside of normal business hours: by phone call or text message, providing appointment reminders, information about how to take medication and care for general health, and to offer general encouragement or support. While participants believed access to healthcare providers was important for people experiencing mental health problems, one participant highlighted the importance of being able to refer patients to mental health professionals, as needed.

Participants noted limited privacy, high clinic volumes, and challenges with patient flow as barriers to mental health help-seeking in HIV care settings. Participants described that the physical environment at the facilities was not conducive to sharing personal information because space constraints limited confidentiality and privacy. Concerns that others may overhear sensitive conversations prevented participants from disclosing mental health concerns. Additionally, high clinic volumes resulted in long wait times, another barrier to care, especially for those who were employed and unable to take time off work. One participant explained how long or unknown wait times were a deterrent:

*“There are some doctors you go to see that are like ‘I’m busy’ or ‘You cannot see the doctor now. Go and come back maybe in four hours or in three hours or in ten hours.’ You know, all of that. You will never make me feel like coming to see the doctor” (Female, 22, reported MH symptoms).*

To facilitate mental health help-seeking, participants recommended addressing “bottlenecks”, and establishing “protocols”, and “procedures” to streamline patient flow, reduce wait time, and make it easier to book an appointment.

#### Economic vulnerability, transportation barriers, and mental health help-seeking.

Limited transportation and financial resources were barriers to healthcare access. Six clients across all facilities said not being able to afford the fees healthcare facilities requested or the cost of transportation to health facilities impeded access to care:

*“I could arrive at the hospital and they would tell me that in order to see the doctor, I have to pay for the consultation first, and so forth, and so on. And if you can’t afford it, what do you do?” (Female, 28, MH symptoms not reported).*

Transportation-related barriers included the cost of transportation, the distance and time it took to travel to the facility, and not having friends or family who could take them to the facility. Transportation-related challenges were exacerbated for those living in Cameroon’s Anglophone region, where ongoing conflict restricted participant mobility [33].

Economic vulnerability also impacted participants’ mental health. Participants reported that unemployment and limited financial resources led to sadness and stress distinct from the stress they experienced as related to living with HIV. Inadequate financial resources were frequently mentioned in relation to food insecurity, which was several participants’ most pressing need. Some participants viewed financial instability as a direct consequence of living with HIV because low energy levels affected their ability to maintain employment. Participants also discussed how mental health affected their financial stability:

*“When I’m feeling depressed, I don’t work. I don’t do anything. I just want to stay alone. Now me staying alone definitely I may be alone for about a month or two weeks or so. And in that period of time, I may not have been able to do anything financially to support myself.” (Female, 22, reported MH symptoms)*

Some participants indicated that asking others for help to meet their material needs was associated with feelings of shame, which hindered help-seeking and further compounded mental health challenges.

### Preferred HIV care provider roles to support the mental health of PWH

#### Treat clients with “love and care”.

Several participants recommended that providers consistently treat their clients with “love and care” because they wanted to “feel at home” when receiving HIV care. Many described the importance of providers listening to patients and asking questions to learn how they can best support their clients. If providers understand their *“reason for sadness, then they can easily handle those sad situations of their clients or patients” (Female, 38, MH symptoms not reported)*. To encourage openness, participants wanted their providers’ “manner of approach” to be cordial, friendly, kind, polite, welcoming, and nonjudgmental. Some participants noted that not all PWH would feel comfortable disclosing sensitive information without prompting, that *“they will not express themselves if you [do] not ask” (Female, 38, MH symptoms not reported)*. Participants recommended providers proactively ask about their clients’ relationships and relationship quality, material needs including food insecurity, and how they are coping with the Anglophone crisis. Once the provider better understands the struggles their patients are facing, they can provide more targeted advice and resources.

HIV care providers were trusted sources of social support. Some participants characterized their providers as a “second family” or someone who understood them more than anyone else. For some PWH, HIV care settings were a preferred location to receive mental health care because providers and staff already knew their clients’ HIV status and had established relationships with clients through regular HIV care visits. Some participants noted that visiting their HIV care provider when they were depressed or “had nerves” helped relieve feelings of sadness or depression. Further, positive experiences with providers during routine ART appointments could instill confidence, establish trust, and facilitate future help-seeking.

#### Promote acceptance of HIV status.

Participants believed HIV care providers played an important role in helping PWH accept their HIV status and directly linked acceptance of their HIV status to their mental health. Participants often reported feeling stressed and overwhelmed soon after being diagnosed with HIV due to the chronic nature of HIV, the need for lifelong ART, and persistent HIV-related stigma in Cameroon. HIV treatment was commonly reported as burdensome for participants. Despite being “very, very appreciative” of HIV treatment advances and increased access to ART in Cameroon, participants felt they had less “freedom” compared to people without HIV due to the chronic nature of HIV and the need to manage their ART and attend regular HIV appointments. Participants also communicated they felt lonely and isolated soon after their HIV diagnosis. Some felt obligated to “live in hiding” and take ART in secret to minimize HIV-related stigma and avoid “exposing” their HIV status to others. Being treated with kindness and compassion by HIV care providers fostered greater acceptance of their diagnosis and development of a new sense of normalcy as an individual living with HIV. Participants reported that receiving clinical guidance on HIV management improved their mental health. Clients articulated the advice they received from their HIV care providers *“strengthens you, it brings you back to reason” (Female, 36, MH symptoms not reported)*, *“makes me feel […] better and relaxed” (Female, 36, MH symptoms not reported)*, *“help[s] me calm down and be myself” (Female, 38, MH symptoms not reported)*, and *“you get back a taste for life” (Female, 28, MH symptoms not reported)*. Participants wanted providers to *“try to make them understand that living with HIV is not the end of the world for you and try to make them understand that you still have better days ahead and you still have a life to live.” (Female, 28, MH symptoms not reported).* Participants appreciated reassurance from HIV care providers about the effectiveness of ART and advice around family formation. Learning about safe ways to pursue their goals for family formation and live a “normal” life “galvanized” participants, gave them confidence and hope, and helped them accept their diagnosis.

#### Economic empowerment and material support.

Participants frequently discussed a desire for HIV clinics to provide opportunities for economic empowerment and to address their material needs. Participants across all clinics desired stable employment or job training to “generate income,” “live a better life” and “be encouraged.” Participants explained that having a stable job or stable income can improve overall mental health and well-being:

*“If I could get a stable job, I would leave in the morning and come back at night, and, with the little family I have, I know I would be wealthy. Even that disease I have, maybe it [stress] will just go away.” (Male, 35, reported MH symptoms)*.

Participants explained that being able to take care of oneself and one’s family would alleviate stress associated with living with HIV. Several participants equated having financial resources to “feeling free”, whether it was freedom to obtain necessary medical treatment for opportunistic infections, or to simply live life doing *“what I want, when I want, and how I want” (Female, 28, MH symptoms not reported).*

Participants indicated receiving direct financial support could help them meet a variety of needs, such as purchasing food for themselves or their families, paying their children’s education expenses, or paying for transportation to health care facilities. One participant described how if they were to receive financial assistance from the healthcare facility it would “really boost morale,” make them feel cared for, and they would feel motivated to “work hard and take care of [themself]”. Another participant mentioned the small loans they received from their doctor helped ensure they could visit the facility as needed.

## Discussion

In this study, we explored the experience of mental health help-seeing at HIV care settings and preferred HIV care provider roles to support the mental health of PWH among a sample of PWH in HIV care in Cameroon. Overall, participants reported that they were less likely to seek support from medical providers for mental health compared to physical health needs. However, previous positive contact with providers facilitated mental health help-seeking among participants. Health facility characteristics, such as limited provider availability, concerns about privacy and confidentiality, and high clinic volume were noted as barriers to mental health help-seeking at HIV facilities. Participants consistently reported that they desired positive, caring interactions with HIV care providers and clinic staff and noted that providers could play a critical role in supporting the mental health of PWH by helping them accept their HIV diagnosis and address HIV-related stigma. Participants also noted that directly addressing material hardship through economic empowerment activities or direct financial support also has the potential to reduce stress and enhance their mental health.

Overall, participants did not view HIV care providers or staff as a primary source of mental health-related support. This is consistent with previous research both with this sample and others that indicates that individuals were more likely to seek mental health-related support from informal rather than formal sources [34]. Quantitative research with the cohort of PWH in Cameroon from which this qualitative sample was drawn found that among PWH in Cameroon with symptoms of depression, anxiety, PTSD, or unhealthy drinking less than one-quarter (24%) had ever sought help from a formal source, (e.g., HIV care provider, mental health specialist) while almost half (46%) had sought help from an informal source (e.g., family, friends, traditional healers, religious leaders) [34]. Reluctance to view HIV care providers as a potential resource for mental health support may be informed by the limited mental health resources currently provided in these HIV care settings and limited mental health training of many HIV care providers in Cameroon. Limited formal mental health help-seeking may also be influenced by mental health-related stigma and limited community awareness of mental health disorders and the effectiveness of evidence-based mental health treatments [35]. While research focused on mental health help-seeking among PWH in sub-Saharan Africa remains limited, perceived need and effectiveness of mental health care and mental health-related stigma have been identified as key barriers to mental health help-seeking in Uganda and Nigeria as well as other low-resource settings [36–39]. A systematic review of the relationship between mental health-related stigma and help-seeking found that internalized and treatment-related mental health stigma were negatively associated with mental health help-seeking [35]. Similarly, a study with people with mental illness in Ethiopia found that delayed mental health help-seeing was associated with believing mental illness was shameful [40]. Further, a systematic review of mental health help-seeking in Ethiopia found that formal mental health help-seeking was positively associated with believing that mental illness requires treatment and awareness of the availability of mental health treatment [41]. Integration of mental health screening and treatment or referral in these settings should be paired with community psychoeducation about mental health disorders and evidence-based treatments as well as mental health-related stigma-reduction activities. While not specifically targeted to PWH, mental health-focused awareness raising campaigns in Uganda and Nigeria have been associated with increased mental health help-seeking [36,42,43]. The extent to which stigma-reduction activities increase acceptability and uptake of mental health screening and referral or treatment in HIV treatment settings warrants further investigation.

Participants also consistently emphasized the importance of previous positive interactions with HIV care providers in facilitating disclosure of mental health concerns to HIV care providers. Having a friendly, nonjudgmental rapport with HIV care providers made it easier for PWH to discuss sensitive, potentially stigmatizing topics with their providers, including mental health concerns. The role of patient-provider communication in fostering mental health-related help-seeking and disclosure should be further explored. Promising strategies to foster trusting, open, warm relationships between PWH and HIV care providers including active listening, demonstrating interest, and allocating adequate time to discuss sensitive, potentially stigmatizing topics should be implemented and evaluated. These recommendations are aligned with suggestions for improved patient-provider rapport for PrEP delivery in Kenya [44]. Similarly, a qualitative study with PWH new to HIV care in the United States found that providing reassurance to patients, encouraging patients to ask questions, and avoiding judgmental language and behaviors fostered patient-provider trust and reduced patients’ anxiety [45]. The extent to which improved patient-provider communication may increase disclosure of mental health-related concerns or uptake of mental health screening, treatment, or referral warrants further exploration.

The physical structure of HIV treatment centers was also noted as influencing mental health help-seeking in these facilities. Ensuring privacy and confidentiality, reducing wait times, and ensuring reasonable caseloads for HIV care providers are strategies that may enhance mental health help-seeking and disclosure of mental health needs and concerns in these settings. Qualitative interviews focused on barriers and facilitators to integrating mental health screening and treatment in these settings were conducted with HIV care providers in these HIV clinics [46]. Similar to findings with PWH, HIV care providers interviewed in these settings indicated that inadequate physical space and high clinic volume limited their ability to screen PWH for mental health disorders [46]. To foster mental health help-seeking, the physical structure of HIV facilities needs to be enhanced to promote privacy and confidentiality and to allow adequate time for discussion of sensitive and stigmatizing topics.

Participants viewed HIV care providers as playing a key role in facilitating greater acceptance of their HIV status and managing internalized HIV-related stigma which, in turn, may foster better mental health of PWH. HIV diagnosis can serve as an acute stressor for PWH. Access to supportive, caring HIV care providers may facilitate coping and acceptance of one’s HIV diagnosis and reduce internalized stigma and mental health concerns. Providers also can play a key role in providing counseling on the effectiveness of ART, the importance of viral suppression, and the ability to live a long, healthy life with HIV if sustainably virally suppressed. Given that HIV-related stigma remains pervasive in many global settings, for some PWH, HIV care providers may be the only individuals aware of their HIV diagnosis and thus, the only source of support available as one navigates a new diagnosis. Providers can also support HIV disclosure to loved ones which may reduce isolation, increase social support, and reduce mental health symptoms [47,48]. Strategies for HIV care providers to more effectively foster acceptance and reduce internalized HIV-related stigma should be explored.

The relationship between material hardship and mental health was noted by study participants. Quantitative research with PWH has similarly found food insecurity and other forms of material hardship to be consistently associated with symptoms of depression and anxiety throughout sub-Saharan Africa including Kenya, South Africa, and Ethiopia [49–51]. The directionality of this relationship remains unclear and, indeed, emerging evidence suggests the relationship may be bidirectional in which symptoms of common mental disorders, such as depression and anxiety, lead to economic vulnerability and economic vulnerability leads to worsening depression or anxiety [52]. Future research should examine the extent to which economic empowerment interventions, alone or in combination with evidence-based mental health treatment improve the mental health and HIV treatment outcomes of PWH. Longitudinal qualitative research of a livelihood intervention with PWH in Kenya found that improvements in symptoms of depression and anxiety following the intervention occurred through several mechanisms including increased food security and reduced economic vulnerability, increased physical activity, and improved sense of self [53].

This study has limitations worth noting. Data were collected from PWH at three urban HIV clinics in Cameroon. As such, the experiences of PWH in rural settings in Cameroon may be meaningfully different. However, it also is important to note that to avoid HIV-related stigma and disclosure of one’s HIV status, PWH in Cameroon often travel substantial distances to receive HIV care in communities far from their home. It is possible that participants in this study lived in rural areas despite receiving HIV care in urban areas. In addition, data were collected from PWH who had initiated HIV care. Findings may differ among PWH who have been diagnosed but have not yet initiated HIV care. Further, participants were screened for symptoms of common mental disorders, including depression, anxiety, and PTSD, as well as unhealthy drinking. Perspectives on mental health help seeking may be different among people with and without symptoms of serious mental illness.

Overall, this sample of PWH in Cameroon reported that mental health-related help-seeking was limited in HIV care settings, but identified strategies that have the potential to enhance both mental health related-help seeking in HIV care settings and may improve the mental health of PWH. The extent to which strategies to enhance patient-provider communication, foster acceptance of one’s HIV status, and address the material needs of PWH enhance mental health help-seeking and improve mental health should be evaluated.

## Supporting information

S1 Text In depth interview guide. (DOCX)
