# Supplementary material for: Mental health-related help-seeking and the role of HIV care providers: A qualitative study with people with HIV in Cameroon
Source: PLOS Glob Public Health. 2025 May 15;5(5):e0004597. doi: 10.1371/journal.pgph.0004597 (PMC12080824; doi:10.1371/journal.pgph.0004597)
Supplement: S1 Text — (DOCX) [file pgph.0004597.s001.docx]

**In-depth Interview Guide**

The purpose of this interview is to help doctors and healthcare providers understand how best to integrate mental health screening and treatment into HIV care. Your opinion is important to better understand what things might make integrating mental health screening and treatment easier for people with HIV. Remember that you never have to answer any question if it makes you uncomfortable.

1. Many people with HIV experience emotional problems, sadness, or depression. Why do you think this is?
2. Have you experienced emotional problems, sadness, or depression? What affects the way you feel?
3. Do you think the way you feel makes it easier or harder to see the doctor? How have providers helped you with your emotional problems, sadness, or depression?
4. Tell me about the last time you felt sad. What was it that made you feel sad recently?
5. What things do you think health care providers need to know about caring for people with HIV who experience emotional problems, sadness, or depression?
6. What helps you to see the doctor when you feel sad?
7. What makes it harder to see the doctor when you feel sad?

1. What do you think most people think about people with emotional problems, sadness, or depression?
2. What would make it easier or more comfortable for people with HIV to get help if they had emotional problems or felt sad or depressed?
3. What do you think can be done to improve the health and well-being of people with HIV?
4. Is there anything else that we didn’t talk about that you want to share?

Thank you very much for your time and responses.
